# Supplementary material for: Retrieval augmented generation for large language models in healthcare: A systematic review
Source: PLOS Digit Health. 2025 Jun 11;4(6):e0000877. doi: 10.1371/journal.pdig.0000877 (PMC12157099; doi:10.1371/journal.pdig.0000877)
Supplement: Attachment — Submitted filename: Response to Reviewers.pdf [file pdig.0000877.s004.pdf]

Rebuttal

We would like to thank the reviewers for their valuable feedback on our manuscript titled *"Retrieval Augmented Generation for Large Language Models in Healthcare: A Systematic Review.* We appreciate their constructive suggestions, which have helped us enhance the clarity and rigor of our work. Below, we provide a point-by-point response to each of the reviewers' comments, along with the corresponding modifications to the manuscript.

Reviewer #1 Comments and Responses:

Comment 1: The notation of "Naïve RAG" which is used by the authors is not very common in the literature. Could the author explain why they call it Naïve RAG? I think this should be changed. Similar story of the term "Advanced RAG." How do you define "Advanced"?

Response: We acknowledge the concern raised regarding the terminology. The term "Naïve RAG" was used to describe basic RAG implementations that do not incorporate additional optimisation techniques such as chunk optimisation, retrieval filtering, or post-retrieval ranking. Likewise, "Advanced RAG" was used to categorize implementations that employ sophisticated retrieval and augmentation mechanisms. We choose to use these terms as they are commonly used in the RAG literatures as defined by Gao et al. (2024).

Gao Y, Xiong Y, Gao X, Jia K, Pan J, Bi Y, et al. Retrieval-Augmented Generation for Large Language Models: A Survey; 2024.

We provide justification that we discuss advancement of RAG in healthcare using RAG framework as defined Gao et al.

On page 8, line 203-208: "A recent review outlined the progression of RAG technologies and their impact on tasks requiring extensive knowledge [16]. Three developmental approaches within the RAG framework were outlined: Naive, Advanced, and Modular RAG, each representing a successive improvement over the one before. In the context of this review, we discuss the RAG techniques used in healthcare by grouping them into these three categories."

However, we understand that they may not be common to everyone. As such we make the following changes.

Manuscript Changes:

Page 8, RAG Overview: Made the following changes.

- Page 9, line 210: We changed sentence defining Naive RAG: Naive RAG is one of the earliest techniques employed to ground LLMs to generate text using relevant information retrieved from external knowledge sources.

  New sentence: "Naive RAG is a basic implementation of RAG and one of the earliest techniques employed to ground LLMs to generate text using relevant information retrieved from external knowledge sources".

- Page 14: We added: definition on Advanced RAG: **Advanced RAG is an improvement of Naïve RAG by incorporating additional pre and post retrieval steps to refine information from the knowledge sources. These additional stages improve the quality and accuracy of the information retrieved, ensuring it blends seamlessly with the model's output.**

Comment 2: Given the very fast-paced nature of this domain, especially RAG in the past months, I propose the authors consider the literature up until September 2024 instead of March 2024, as there has been further work recently published such as (1-3).

(1) Freyer, Oscar, et al. "A future role for health applications of large language models depends on regulators enforcing safety standards." The Lancet Digital Health 6.9 (2024): e662-e672.
(2) Arasteh, Soroosh Tayebi, et al. "RadioRAG: Factual Large Language Models for Enhanced Diagnostics in Radiology Using Dynamic Retrieval Augmented Generation." arXiv preprint arXiv:2407.15621 (2024).
(3) Luo, Ming-Jie, et al. "Development and evaluation of a retrieval-augmented large language model framework for ophthalmology." JAMA ophthalmology (2024).

Response: We agree with the reviewer that the field is evolving rapidly. We have extended our literature search to include relevant studies published up until February 2025, incorporating recent advancements, including those referenced by the reviewer.

Manuscript Changes:

- Page 3, Methods: Updated the literature search timeframe from January 2020 to February 2025.
- In table 3, in results section: Added newly identified studies.
- Revised the discussion accordingly.

Comment 3: Table 1 could be integrated into the text and removed from the table list.

Response: We have integrated the information from Table 1 into the text while ensuring readability.

Manuscript Changes:

- Page 3, Methods: Integrated Table 1 into the text.

Added: Inclusion criteria

- Language of articles: English
- Articles published between January 2020 and February 2025
- Articles covers: RAG and LLMs in the medical domain.

Comment 4: Have the authors obtained permission from the owner of Figure 2 to include it in the manuscript? Furthermore, the fonts are very small in that figure and not easily readable.

Response: To clarify, we did not copy Figure 2 from the publication mentioned in the caption. We created it from scratch, taking inspiration from a visualization by Gao et al. (2024). The wordings and example provided are our own. We removed the text, Adapted from" from the caption for the sake of clarity. Additionally, we have increased the font size to improve readability.

Manuscript Changes:

- Page 8, Figure 2 remove "adapted from" text from the caption.
- Updated Figure 2 with larger fonts.

Comment 5: A better representation could be chosen instead of Table 5. Currently, it is difficult to read through it.

Response: We have placed Table 5 with Figure 3 to improve readability and clarity.

Manuscript Changes:

- Page 22, Figure 3 to enhance readability.

Comment 6: Figure 3 could be improved: Fonts should be larger, and subfigure A could be integrated into subfigure B.

Response: We have increased the font size in Figure 3 (Now Figure 4). Difficult to merge the two figures. Thus, we kept them separate sub-figures.

Manuscript Changes:

- Page 24, Figure 4: Updated to enhance clarity.

Reviewer #2 Comments and Responses

Comment 1: It is not clear who performed the screening of the articles and if independent screening was performed.

Response: We appreciate this observation. We have clarified the screening process.

Manuscript Changes:

- In line 87 on page 5, Methods: Added a statement: **Screening and data extraction were carried out by multiple reviewers following inclusion and exclusion criteria**

Comment 2: A quantitative analysis of the performance of RAG vs native LLMs will be helpful in contextualizing the utility of RAG.

Response: We acknowledge the importance of quantitatively comparing RAG-based approaches with native LLMs. Even though it is difficult to compare because of the different metrics used. Throughout the results discussions and especially in the outcome column in table 3, we report quantitatively how the different RAG implementations have outperformed baseline models.

For example:

"RAG achieved 98% accuracy providing reliable accuracy"

"Achieved an average performance improvement of 20.72%"

"Improved performance with RAG over baseline models"

"RAG improved the accuracy by an average 39.7%"

Manuscript Changes:

- Page 20, Results section: In addition to the many statements comparing results over the studies we reviewed. We added new statements discussing quantitative comparisons between RAG and native LLMs.

For example, "Woo et al. [109] has demonstrated that incorporating AI agents into a previously RAG-augmented LLM improved the accuracy of GPT-4 to 95%."

Conclusion

We have carefully addressed all reviewer comments and made the necessary revisions to improve the clarity and depth of our manuscript. We believe these changes significantly enhance the manuscript’s quality and appreciate the reviewers' valuable input.
